# Curability of Uterine Displacement

**Published:** 1881-02

**Authors:** P. O’Connell

**Affiliations:** Sioux City, Iowa


### ARTICLE III.

CURABILITY OF UTERINE DISPLACEMENT. By P. O'Connell, M.D., Sioux City, Iowa.

At a recent meeting of the Obstetrical Society of London, Dr. Graily Hewitt read a paper on Uterine Displacement. During the discussion of the paper some very strange opinions were expressed, and coming from old and experienced teachers are apt to mislead. Dr. Matthews Duncan said: "The treatment of displacement without descent, whether it was desirable or not, was an utter failure; and displacement was never cured, in the sense that the uterus afterwards remained in place without a pessary." With all due respect to the opinion of Dr. Matthews Duncan, I beg to say that the treatment of uterine displacement, in my hands and in the hands of my professional friends in Chicago, has been attended with most gratifying success; and, *in the sense that the uterus has remained in situ without a pessary*, the displacement *was absolutely cured.* All the distressing, and in some cases, dangerous symptoms passed away; the bowels became regular; menstruation became normal where before it was abnormal; the general health was entirely restored.

Until I read the astounding asertion of Dr. Matthews Duncan, I did not suppose there was any doubt of the curability of uterine displacements with pessaries. Fifteen years ago, displacements of the uterus were not supposed, except by a few, to give rise to serious trouble. Even to-day so high an authority as Dr. Duncan declares, "It would be nearer the truth to say it" (displacement) "had little or no importance." Mr. Thornton agreed with Dr. Duncan, and believed that cases complicated by descent "alone gave trouble or required aid;" while Dr. Savage said, "pessaries without stems merely kept the uterus out of the vagina." I believe it was the late Dr. Washington L. Atlee who declared that while he withdrew many pessaries, he never had inserted one, and had no faith in them. I am acquainted with a very distinguished physician and teacher in Great Britain who makes no pretense to a knowledge of gynæcology, has no faith in pessaries, and treats uterine displacements by simple replacement daily with his finger. Such sweeping condemnation of pessaries without at least a trial, is unphilosophical. Surely neither of the gentlemen named will say that epilepsia gravior and long-continued menorrhagia are of "no importance," or should go untreated. And yet I have encountered these conditions, as the result of the displacement, in the cases recited below, and there *was no descent of the uterus.* As ideas have changed on the utility of pessaries, so, too, the strong opinions now held and expressed by very distinguished writers, with regard to the incurability of displacement with or without descent, may have to be radically changed ere long. Such dogmatic assertions by well-known teachers sometimes acquire peculiar importance for the general practitioner when, in the hands of dishonest and evil-disposed parties, they are employed to sustain a suit for malicious malpractice. Hence I consider it a duty incumbent on those who are in a position to contradict such teachings to do so.

Unwilling to occupy much of your valuable space, I offer very short notes of a few cases *cured* in every sense of the word. While all were patients in private practice, all were obliged to do pretty hard work either as assistants in shops or in the management of their homes and families. All were permitted, without restriction, to attend to their customary duties after the insertion of the pessary, and instructed to go out of doors daily for exercise. I confine no woman to the house after the insertion of a suitable pessary.

The few cases here given are of retroflexion alone, and the conclusions deduced from them apply equally to cases of the other displacements. The instrument employed in all the cases, save one, was that known as Smith's modification of the Hodge pessary. No woman was pronounced *cured* until it was ascertained by actual trial that she could dispense with the instrument. This is done by removing the pessary and permitting the patient to discharge all her duties as before. If, after the lapse of a few weeks, the uterus is found in normal position, as determined by the finger and the sound, the pessary is not re-introduced. My observation of these women extended over one year, the shortest, to four years, the longest, period after the removal of the pessary; and in no case was the displacement reproduced. Surely, a woman who has freely discharged all her duties for an entire year after the removal of a pessary, without any return of the displacement, is entitled to be called *cured.* If she cannot be called cured after that time, then I admit uterine displacements are incurable. I have seen many who are really incurable no matter what plan of treatment be employed, even while the uterus is free and easily raised into position. This is the experience of every man who deals with diseases of women. Is it, then, right to conclude that because some are, all must be incurable? The conclusion is absurd. The want of due care in selecting a pessary, in form and size, to suit each individual case, is also a cause of the distrust of pessaries. I have seen pessaries turn completely upside down or fall in front of the cervix when they should be the other way. The man who should employ so ill-fitting an instrument can meet with nothing but disappointment, and is pretty sure to be the first as well as the loudest in condemning pessaries.

In two instances only of my cases, case I and VII, was the displacement complicated by descent of the uterus, and in neither case did the descent cause any particular trouble.

Case I. Mrs. B, aged twenty-six, had three children; general health considerably below par; had an abortion in third month of pregnancy, eighteen months before coming under my care.

Doubtless the flexion happened then. Has prolapse, sometimes down to the vulva, owing to enlargement of the body of the uterus. Menses scanty. On July 27, 1875, I inserted a pessary, which she wore eight months continuously. On March 29, 1876, I withdrew the instrument against her wish, and never after re-inserted it. Menstruation became normal, and her general health entirely restored.

Case II. Mrs. K., aged thirty; one child. Flexion exists, most likely, one year. Patient so weak as to be obliged to resign her situation in a dry-goods establishment. During defecation, the contents of the bowel caused such intense suffering in the retroflexed fundus, that she voluntarily induced obstinate constipation. July 5, 1876, inserted a pessary, and withdrew it November 5, 1876. Her health was entirely restored, and she was able to resume her situation.

Case III. Mrs. E., aged twenty-two, married fourteen months; not yet pregnant. Eighteen months ago menstruation became irregular, with pain in the uterus, during defecation, the day before the flow appeared. Had suffered pretty severely from menorrhagia, for which she consulted me. Is much exhausted. Inserted a pessary, November 27, 1876. It was removed twice for trial during 1877, and withdrawn finally, February 28, 1878. Menstruation became normal; health entirely restored.

Case IV. Mrs. C., aged thirty; five children; had a miscarriage at seven and a half months, due to syphilitic infection by her husband. She suffered from menorrhagia and was extremely debilitated. Flexion exists six months. On February 8, 1877, inserted a pessary, and removed it February 14, 1878. Menses became normal, and health good. Had one child, at term, in March, 1880.

Case V. Miss D., aged twenty and a half, is quite anæmic and has a severe eczema. Three years ago amenorrhœa and dysmenorrhœa began; possibly flexion began then and was the cause of the menstrual difficulties. Inserted a pessary February 11, 1877, and removed it February 6, 1878. The anæmia disappeared and menstruation became normal.

Case VI. Mrs. O., aged thirty-seven, never pregnant; health fair, and menses regular. Had acute endo-metritis; after its cure the uterus was found flexed, the flexion being only of a few days duration. Eight years ago, this lady had suffered for some months from acute retroflexion, caused by constipation. At that time she was under another physician's care, who did not suspect any uterine mischief for a few months, during which she had several attacks of epilepsia gravior. When the flexion was cured then by means of tents and a pessary, the epilepsy ceased and did not recur. Inserted a pessary February 4, 1878, and removed it on February 22, 1878.

Case VII. Mrs. L., aged twenty-seven, four children; nursing. Has prolapse, and much leucorrhœa; debilitated and hysterical. Flexion exists a year. Inserted a Hodge pessary on June 5, 1878, and removed it December 15, 1878, as she had "quickened," pregnancy having taken place during the use of the pessary.

Case VIII. Mrs. W. aged forty, a very large, awkwardly fat woman, and quite debilitated. Is twenty years married, but never pregnant. Suffered from frequent micturition for the past fourteen months. There is subacute vaginitis. Doubtless the flexion is as old as the vesical irritation. Menses regular. Inserted a pessary January 14, 1879, and removed it April 4, 1879. The vesical irritability passed away with the correction of the flexion.

With this case, I will close my communication, already, I fear, too long. Were it necessary, I could cite other cases equally interesting and important. From these cases I hope it is clear that *displacement* of the womb possesses *much* importance, and that, too, when there is no *descent* of the organ. In addition to these cases, an unmarried lady, a music teacher, came under my care quite recently, suffering from retroflexion with slight descent. The flexion was recognized six years ago, and may be of ten years duration. During that time she is afflicted with severe and very irregular epilepsy. A suitable pessary kept the uterus *in situ* after it was elevated. While the pessary was worn she had no epileptic paroxysm. On the removal of the pessary, the attacks recurred. The insertion and removal of the pessary was tried sufficiently often to prove that the flexion of the uterus was the cause of the epilepsy. She still wears her pessary; the result, therefore, cannot be foretold so far as the effect on the flexion is concerned. Even if the flexion must remain uncured, and I suspect it is incurable in this case, the benefit of the pessary is well displayed by warding off the epileptic paroxysms, a matter of the very greatest, in fact incalculable, benefit to this lady.

I feel warranted in deducing the following conclusions from my experience:

1. Displacements of the uterus are, in the great majority of women, serious.

2. That descent adds but little, in many cases, to the gravity of the trouble.

3. That displacements can be cured, by suitable pessaries, in the sense that the pessary may be discarded after a time, and the uterus remain *in situ* without the support the pessary afforded—that the displacement is absolutely cured.

